# Genetic and cross-species single-cell analyses prioritize CD83 in antigen-presenting cells as a candidate therapeutic target in asthma

**DOI:** 10.3389/fimmu.2026.1859076

**Published:** 2026-07-13

**Authors:** Guojun Qian, Qiaoyi Xu, Xuejiao Zhu, Yiying Tao, Xibing Ding, Hao Zhu, Song Zhang, Qionghui Zhan, Saihong Xu, Yan Song, Qin Hu, Hongwei Fang, Yinghui Fan, Jie Tian, Weifeng Yu

**Affiliations:** 1 Key Laboratory of Anesthesiology (Shanghai Jiao Tong University, Ministry of Education), Department of Anesthesiology, Ren Ji Hospital, Shanghai Jiao Tong University School of Medicine, Shanghai, China; 2 Department of Critical Care Medicine, Ren Ji Hospital, Shanghai Jiao Tong University School of Medicine, Shanghai, China; 3 Department of Anesthesiology, The Second Affiliated Hospital of Soochow University, Suzhou, Jiangsu, China; 4 Department of Laboratory Medicine, Ren Ji Hospital, Shanghai Jiao Tong University School of Medicine, Shanghai, China; 5 Department of Neurosurgery, Ren Ji Hospital, Shanghai Jiao Tong University School of Medicine, Shanghai, China; 6 Department of Pain Management, Ren Ji Hospital, Shanghai Jiao Tong University School of Medicine, Shanghai, China

**Keywords:** allergic inflammation, antigen presentation, asthma, CD83, immunomodulation, mendelian randomization

## Abstract

**Background:**

Asthma remains a heterogeneous inflammatory airway disease, and current therapies, including biologics, benefit only selected patient subsets. We aimed to identify genetically supported therapeutic targets with clear mechanistic and translational relevance in asthma.

**Methods:**

We integrated cis-eQTL and cis-pQTL instruments for 585 druggable genes with asthma genome-wide association data from FinnGen, EBI, and UK Biobank, comprising more than 1.1 million participants in total, using Mendelian randomization, colocalization, and SMR/HEIDI analyses. We then combined cell-type-specific single-cell eQTL Mendelian randomization with lung single-cell RNA sequencing from a house dust mite mouse model and public human and cynomolgus monkey single-cell datasets to define the cellular context of the prioritized signal. Phenome-wide association analysis and Connectivity Map screening were used to assess target specificity and nominate candidate compounds.

**Results:**

CD83 emerged as the most consistently supported candidate across the genetic prioritization pipeline. Higher genetically predicted CD83 expression showed a consistent risk-increasing association across independent cohorts, and protein-level analyses together with colocalization provided convergent support for CD83 as the prioritized gene at this locus. Mechanistically, integrating cell-type-specific human genetics with cross-species single-cell transcriptomics localized the signal to antigen-presenting cells, particularly memory B cells and dendritic cells. Across human, mouse, and monkey datasets, CD83-positive antigen-presenting cells were consistently enriched for MHCII antigen-presentation and T-cell activation programs following allergen challenge. Phenome-wide analyses did not identify major genome-wide significant associations outside the asthma signal, and signature-reversal analysis highlighted hydroxyfasudil and prunetin as candidate compounds for further evaluation.

**Conclusions:**

These findings prioritize CD83 in antigen-presenting cells as a genetically supported candidate therapeutic target in asthma. More broadly, they provide a translational framework linking human causal genetics to cell-specific mechanism and therapeutic nomination, and support direct CD83 perturbation studies in airway inflammation.

## Background

Asthma is a heterogeneous chronic inflammatory disease affecting over 300 million individuals, driven by complex pro-allergic immune responses that impose a substantial burden on patients and healthcare systems (1–3). Its clinical presentation and severity are highly variable, with children and women being disproportionately affected (3, 4). Current therapies, such as corticosteroids, provide broad immunosuppression, while newer biologics targeting specific cytokines (e.g., IgE, IL-5, IL-4/13) are effective only in select patient subsets (1, 3–5). This underscores a critical unmet need for new therapies that target upstream immunomodulatory pathways with broad applicability across the asthma spectrum.

Identifying such causal drivers has been a central challenge. While genome-wide association studies (GWAS) have linked hundreds of loci to asthma, translating these statistical associations into validated therapeutic targets remains a major bottleneck (6, 7). To bridge this gap, modern causal inference methods like Mendelian randomization (MR) are essential, as they leverage human genetic variation to dissect causal relationships (8–11). However, to confidently prioritize a gene for therapeutic evaluation in asthma, it is crucial to overcome methodological pitfalls like genetic linkage (12) and pleiotropy (2) and to pinpoint the specific immune cell types where the gene exerts its pathogenic effects.

To meet this challenge, we developed a comprehensive translational pipeline designed to move from genetic signal to actionable biology. Our framework begins by systematically screening a curated set of over 2,500 established druggable genes, integrating genetic instruments for both gene expression (eQTL (13)) and protein levels (pQTL (14)) to identify causal drivers. It then ensures causal fidelity through a rigorous triangulation of colocalization (15, 16) and SMR/HEIDI tests (17, 18). Next, it resolves the immunological context by mapping the target’s action to specific cell types using single-cell and cross-species data. Finally, it establishes a direct path to the clinic by evaluating on-target safety with phenome-wide MR (PheWAS) (19) and identifying candidate small molecule inhibitors via drug repurposing screens (20, 21).

Applying this strategy, we prioritized the immunoregulatory molecule CD83 as a candidate driver of asthma. Our analyses suggest that CD83, acting primarily in B cells and dendritic cells (DCs), may enhance MHCII-mediated antigen presentation and amplify downstream T-cell responses in allergic airway inflammation. We further identified candidate compounds predicted to reverse the CD83-positive APC signature and found no major genome-wide significant phenotype associations in phenome-wide analyses. These results support CD83 as a candidate pathway for further therapeutic evaluation in asthma.

## Methods

### Study design

**Figure 1:**
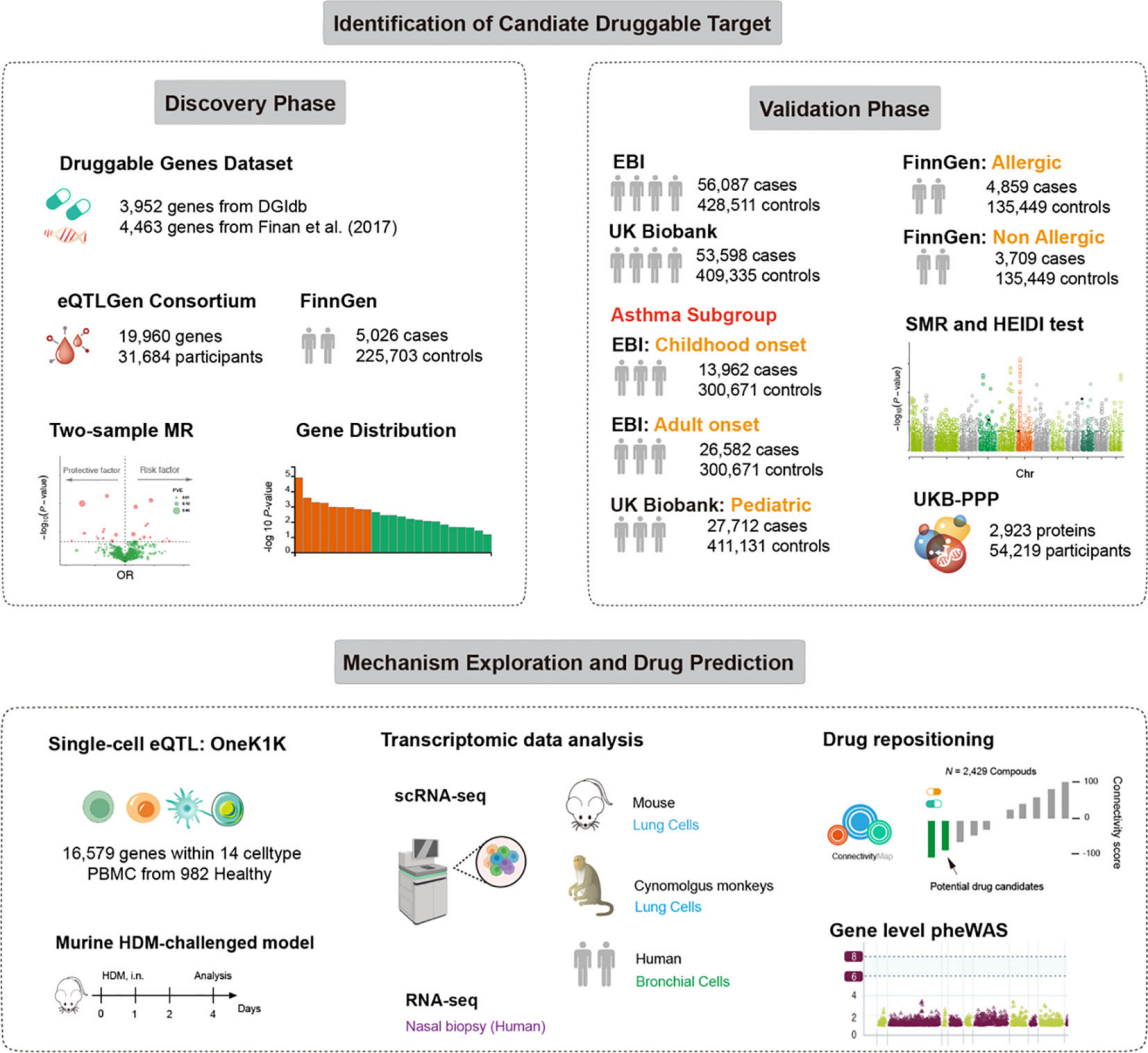
A multi-omic framework for therapeutic target discovery. Our pipeline systematically identifies causal asthma targets via Mendelian randomization (MR) screening of 585 druggable genes using transcriptomic (*cis*-eQTL) and proteomic (*cis*-pQTL) data. Causal loci are then validated for robustness using colocalization, SMR, and HEIDI. We dissect cellular mechanisms using bulk and single-cell transcriptomics (RNA-seq, scRNA-seq, sc-eQTL) and cross-species models. Finally, on-target safety is assessed by phenome-wide MR (PheWAS), and repurposable drugs are identified with the Connectivity Map (CMap).

The analytical workflow is depicted in **Figure 1**. We began by compiling 3,952 potential drug target genes from the Drug–Gene Interaction Database (DGIdb) (22) and 4,463 druggable genes from a research report (23), defining druggable genes as those encoding proteins with known or predicted therapeutic tractability, including approved or investigational drug targets, proteins with ligand-binding or extracellular domains, and genes homologous or functionally similar to established targets. These gene sets were intersected with pQTL data from the UK Biobank Proteomics Project (UKB-PPP) (14) and eQTL data from the eQTLGen consortium (13) to identify overlapping druggable genes. After selecting appropriate eQTL instrumental variables, we conducted MR analyses using asthma data from the FinnGen cohort (24). Genes that met significance thresholds were further validated in replication cohorts from the European Bioinformatics Institute (EBI) (25, 26) and the UK Biobank (26, 27), including subgroup analyses for childhood-onset and adult-onset asthma. Comprehensive sensitivity analyses were conducted to evaluate the consistency of findings across discovery and replication stages. We employed SMR analyses (14–18) to confirm our findings further. To validate the candidate drug target at the protein level, MR analyses were conducted using pQTL data. Colocalization analyses (15, 16) and HEIDI tests (18) were performed to determine whether observed correlations stemmed from common causal variants or LD. Additional verification of target genes involved tests for causal directionality (Steiger filtering), horizontal pleiotropy, and heterogeneity (28, 29). Finally, we evaluated the potential mechanisms and clinical utility of the identified drug target through single-cell eQTL MR analysis, scRNA-seq and bulk RNA data analysis, PheWAS, and in silico drug screening. The study complies with the STROBE-MR guidelines (10), with the checklist provided in **Supplementary Table 1**.

### Exposure data

We obtained eQTL data from the eQTLGen consortium (13), encompassing analyses of 16,989 genes across 31,684 blood samples from healthy European ancestry individuals. Comprehensive information on this dataset is available in their original publication (13). pQTL data were sourced from the UKB-PPP (14), a collaboration between the UK Biobank and multiple biopharmaceutical companies. Single-cell eQTLs were derived from 14 distinct immune cell types using gene expression profiles obtained from 982 participants in the OneK1K cohort (30). The compilation of druggable genes was derived from a prior study that used a computational approach to integrate existing GWAS data, aiming to identify druggable proteins and associate them with known pharmaceuticals (23).

### Outcome data

We utilized three distinct datasets for asthma outcome data. The discovery cohort was sourced from FinnGen Release 10 (24), published in December 2023, comprising 5,026 asthma cases and 225,703 controls. The replication cohorts included data from the EBI (25), with 56,087 cases and 428,511 controls, and the UK Biobank (27), with 53,598 cases and 409,335 controls (**Figure 1**, **Supplementary Table 2**). This multi-cohort approach strengthens the validation of our findings across different datasets. Additionally, for detailed subgroup analyses, outcome data pertaining to childhood-onset and adult-onset asthma, as well as pediatric, allergic, and non-allergic asthma phenotypes, were retrieved from the EBI, the UK Biobank, and FinnGen (**Figure 1**, **Supplementary Table 2**), enhancing the specificity and depth of our investigation.

### MR analysis

MR analyses were performed by using the “TwoSampleMR” R package (version 0.5.7) (30). Prior to analysis, we applied stringent quality control to the single nucleotide polymorphism (SNP) instruments. Strong instrumental variables were selected based on an F-statistic > 10 (32). SNPs were pruned for LD using the 1000 Genomes European reference panel, retaining variants with LD *r (*2*)* < 0.1 *(*31, 32). Steiger filtering (31) was applied to confirm the correct causal directionality.

For genes with multiple instrumental variables, we applied inverse-variance weighting (IVW) (12, 33), weighted median (33), MR-Egger regression (28, 33), Simple mode (31), and Weighted mode (31) to estimate causal effects. For genes with a single instrumental variable, the Wald ratio (31) was used. When results across MR methods were inconsistent, we primarily relied on the IVW estimate. Cochran’s Q statistic was used to assess heterogeneity, and the MR-Egger intercept test was used to evaluate horizontal pleiotropy (28, 33). To adjust for multiple testing, we applied the Benjamini-Hochberg procedure to control the FDR, reporting the adjusted *p*-values (*P_adj;* FDR).

### Colocalization analysis

For genes showing significant MR associations across cohorts, we performed colocalization analyses using the “coloc” R package (version 5.2.3) (15, 16). We examined five hypotheses: H0 (no correlation with either trait), H1 (linked solely to gene expression), H2 (connected only with asthma), H3 (correlations with both traits through different causal variants), and H4 (correlations with both traits arising from a common causal variant). A posterior probability exceeding 0.8 for hypothesis 4 (PP.H4) was regarded as compelling evidence for colocalization, signifying that the identical variant affects both gene expression and the risk of asthma. Such genes were considered potential drug target candidates.

### SMR analysis and HEIDI test

To corroborate the causal relationships uncovered via MR, we performed SMR analyses and HEIDI tests utilizing SMR software (version 1.3.1) (17, 18). The SMR method integrates GWAS and QTL data to distinguish between pleiotropy (a single variant affecting both traits) and linkage (different variants in LD affecting each trait) (18). The HEIDI test assesses whether the association is due to LD rather than pleiotropy (18). In our analyses, a significant SMR association was established with a *P*-value less than 0.05, whereas a HEIDI *P*-value exceeding 0.05 suggested that the observed association was likely attributable to a shared causal variant rather than genetic linkage.

### Murine HDM model and transcriptomic validation analyses

Wild-type mice were intranasally sensitized with 5 µg of house dust mite extract (HDM; Greer Laboratories, Lenoir, NC, USA) in 40 µL PBS on days 0, 1, and 2. On day 4, lungs were harvested and single-cell suspensions prepared (34, 35); live CD45^+^ and CD45⁻ fractions were FACS-sorted and mixed at a 1:1 ratio. Cells were then loaded onto the Chromium Controller (10× Genomics, Pleasanton, CA, USA) to generate single-cell gel bead-in-emulsions using the Single Cell 3′ v3.1 Library & Gel Bead Kit, following the manufacturer’s protocol. Libraries were sequenced on an Illumina NovaSeq 6000 (150 bp paired-end), and data were processed with Cell Ranger (v5.0.0) using default settings. Reads were aligned to the Mus musculus reference genome (Ensembl release 106).

We performed scRNA-seq analysis using the Seurat package (36) (version 5.0.3) in R. The count matrix was combined with clinical information to create a Seurat object. Cells and genes of poor quality were filtered out based on the following criteria: cells with fewer than 200 or more than 3,000 detected genes (nFeature_RNA​<200 or >3,000), cells with mitochondrial gene content exceeding 10% (percent.mt > 10), and cells with total counts exceeding 10,000 (nCount_RNA>10,000) were excluded. The data were then normalized and scaled using default settings in Seurat.

Unsupervised clustering was conducted using a graph-based approach, selecting the top 20 principal components and setting the resolution parameter to 0.4. Clusters were visualized using t-SNE. Cell type classification was carried out using the SingleR package (37) (version 2.2.0) and GPTCelltype package (38) (version 1.0.1), and confirmed by known biological markers as reported.

Differentially expressed genes (DEGs) across each cell type were determined using the FindAllMarkers function, applying thresholds of a log fold change greater than 0.25 and a minimum cell proportion expressing the gene exceeding 0.25, and *P_adj* less than 0.05. Pathway analysis and visualization of DEGs were conducted using the clusterProfiler package (39) (version 4.10.0) in R.

To infer and analyze intercellular communication networks from the single-cell transcriptomic data, we utilized the CellChat R package (40) (v.2.1.2). CellChat was used to systematically quantify signaling probabilities and compare communication patterns between cell populations based on the expression of ligand-receptor pairs. To identify context-specific biomarkers and significant latent factors within the CD83^+^ memory B cell population, we subsequently employed the SLIDE (41) (Significant Latent Factor Interaction Discovery and Exploration) machine learning algorithm.

For focused functional validation, Cd83 was knocked down in lung DCs using a CD11c promoter-driven shRNA approach in the HDM sensitization model. Publicly available scRNA-seq data were used for cross-species validation. We analyzed human bronchial brushings from HDM-challenged asthma patients (GSE164015 (42)) and lung tissue from a cynomolgus monkey model of *Ascaris suum*-induced asthma (GSE213085 (43)). We also used the publicly available nasal biopsy transcriptomic dataset GSE182797 (44) for independent airway validation. The dataset contains baseline nasal biopsy microarray profiles from female adult-onset asthma patients (*n* = 45), IEI patients (*n* = 14), and healthy subjects (*n* = 21). In this study, only adult-onset asthma patients and healthy subjects were used for the asthma-control comparison; detailed cohort information was reported in the original publication (44).

### Candidate drug prediction

We compared the expression signatures of CD83^+^ B cells and CD83^+^ DCs against the entire CMap library of compound-induced profiles in human cell lines (20, 21). Using pattern-matching algorithms, each perturbagen’s signature was scored for both the direction and magnitude of its enrichment relative to our scRNA-seq DEG lists. A positive connectivity score indicates that the compound induces changes similar to those in our query (for example, upregulating genes that are also up in CD83^+^ cells), whereas a negative score reflects an opposite effect.

### PheWAS

We conducted PheWAS using data from the AstraZeneca PheWAS Portal and the PheWeb database to assess the pleiotropic effects and potential adverse consequences of targeting the identified therapeutic gene (19). The original study utilized data from approximately 17,361 binary and 1,419 continuous phenotypes, with participants drawn from the exome sequencing subset of the UK Biobank (19, 45).

### LLM-assisted language editing

ChatGPT (OpenAI) was used only for language refinement after completion of the scientific analyses and interpretation. No AI tool was used for data analysis, figure generation, or scientific decision-making. All AI-assisted edits were reviewed and approved by the authors.

## Results

### Systematic MR prioritizes 12 druggable candidate genes for asthma

**Figure 2:**
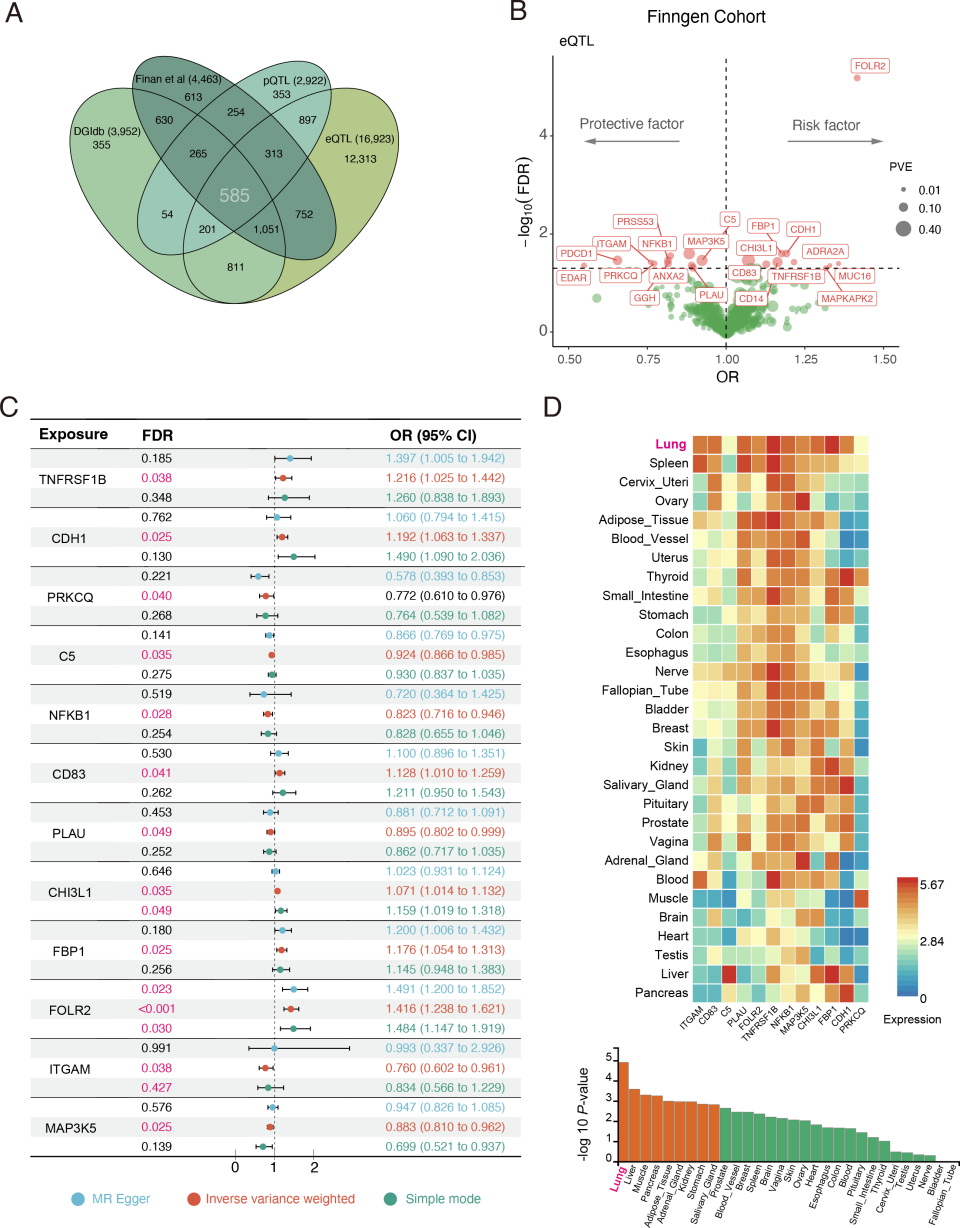
Systematic mendelian randomization screen prioritizes 12 druggable candidate genes for asthma. **(A)** Venn diagram illustrating the prioritization pipeline for druggable genes. From an initial set of 2,532 druggable genes, 585 were selected based on the availability of both transcript-level (cis-eQTL) and protein-level (cis-pQTL) genetic instruments. **(B)** Volcano plot of two-sample MR results from the FinnGen cohort (*n* = 230,729), with the x-axis showing odds ratios (OR) and the y-axis displaying -log10 (FDR). Genetically predicted expression of 29 genes was significantly associated with asthma risk (FDR_IVW or Wald ratio_ < 0.05). Dashed lines indicate the significance threshold. Circle size represents the proportion of variance explained (PVE) by each gene. **(C)** Forest plot showing the 12 core candidate genes that passed all sensitivity analyses. The OR for asthma risk per 1-SD increase in gene expression is shown for three independent MR methods (inverse variance weighted, MR Egger, and simple mode). All 12 candidates showed directional consistency and passed the Steiger directionality test. **(D)** High expression of candidate genes in lung tissue. (Top) Heatmap showing the normalized expression (TPM) of the 12 candidate genes across selected human tissues from the GTEx v8 database. Red indicates higher expression and blue indicates lower expression. (Bottom) Gene set enrichment analysis demonstrating that the 12-gene set is most significantly expressed in lung tissue (*P* < 0.0001, two-sided test). The bar plot shows the -log_10_(*P*-value) for gene set expression across different tissues.

To comprehensively screen for potential drug targets in asthma, we first curated a set of 2,532 established druggable genes by integrating two major databases (22, 23) (**Supplementary Tables 3–5**). To ensure robust causal inference, we refined this gene set by requiring evidence of genetic regulation at both the transcript (cis-eQTL) and protein (cis-pQTL) levels. This dual-evidence filtering yielded 585 high-confidence druggable genes with both transcript-level and protein-level genetic instruments (**Figure 2A**, **Supplementary Table 6**). For these genes, we identified 2,181 high-quality cis-eQTL variants to serve as instrumental variables for MR analysis (**Supplementary Table 7**).

Two-sample MR analysis using data from the large-scale FinnGen cohort (24) (*n* = 230,729) revealed that genetically predicted expression of 29 genes was significantly associated with asthma risk (FDR_IVW or Wald ratio_ < 0.05) (**Figure 2B**, **Supplementary Table 8**). Of these, 14 passed a comprehensive suite of sensitivity analyses (**Supplementary Tables 9**, **10**). We prioritized a final set of 12 core candidate genes, including CD83, PRKCQ, FOLR2 and C5, whose causal estimates were directionally consistent across three independent MR methods and were validated by the Steiger directionality test (**Figure 2C**, **Supplementary Table 11**). Functional enrichment analysis further supported the biological relevance of these candidates to asthma. They were highly expressed in lung tissue and significantly enriched in pathways central to asthma pathophysiology, including inflammatory/defense response, IL-4/IL-2 signaling, and T-cell activation (**Figure 2D**, **Supplementary Figure 1**). Together, these results generated a high-priority list of targets supported by robust genetic and biological evidence, setting the stage for subsequent validation.

### Replication in independent cohorts and SMR/HEIDI tests confirm robust causal effects

**Figure 3:**
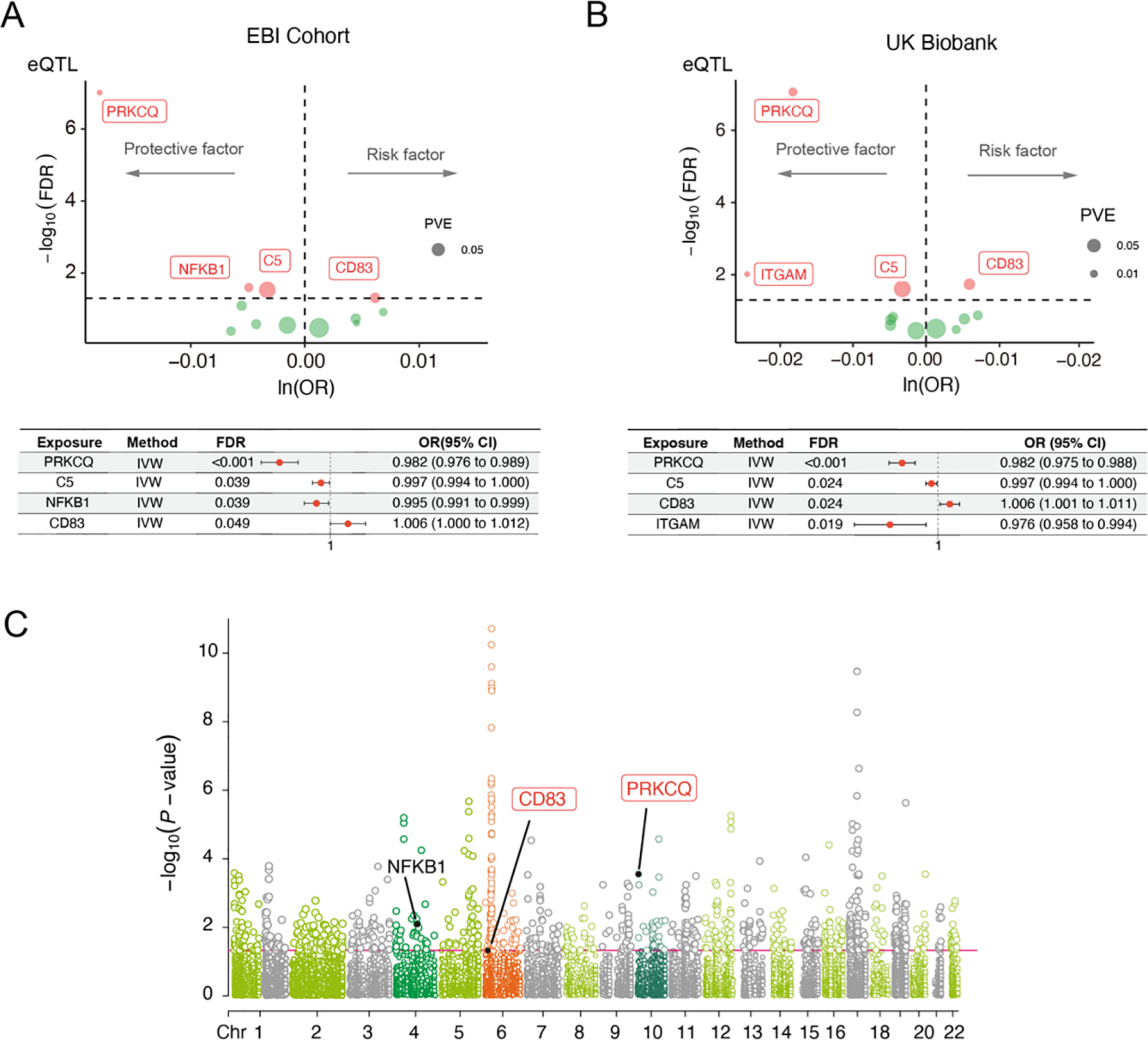
Replication and validation of candidate genes causally associated with asthma. **(A, B)** Replication of causal associations in two independent cohorts. Two-sample Mendelian randomization (MR) volcano plots (top) and corresponding forest plots (bottom) for the **(A)** EBI cohort (*n* = 484,598) and **(B)** UK Biobank cohort (*n* = 462,933). In volcano plots, the x-axis represents the log-transformed odds ratio [ln(OR)] and the y-axis shows the false discovery rate [-log10(FDR)]. Genes with significant causal associations (FDR < 0.05, dashed line) are labeled. The size of each dot represents the proportion of variance explained (PVE). The analyses consistently identified *CD83* as a risk factor and *PRKCQ* and C5 as a protective factor. **(C)** Summary-data-based Mendelian Randomization (SMR) analysis to disentangle causality from linkage disequilibrium. The Manhattan plot shows the SMR association *P*-value for all tested genes. The red line indicates the significance threshold (*P*_SMR_ < 0.05). Labeled genes, including *CD83*, *PRKCQ*, and *NFKB1*, passed both the SMR significance test and the HEIDI test for heterogeneity (*P*_HEIDI_ > 0.05), supporting a direct causal role for the expression of these genes.

To validate these findings, we first performed replication analyses in the independent EBI cohort (*n* = 484,598). These results confirmed that increased expression of PRKCQ (OR = 0.982; 95% CI: 0.976–0.989; FDR_IVW_ = 3.92 × 10^-7^), C5 (OR = 0.997; 95% CI: 0.994–1.000; FDR_IVW_ = 3.89 × 10^-2^), and NFKB1 (OR = 0.995; 95% CI: 0.991–0.999; FDR_IVW_ = 3.89 × 10^-2^) was significantly associated with a reduced risk of asthma. In contrast, increased CD83 expression was associated with an elevated risk (OR = 1.006; CI: 1.000, 1.012, FDR_IVW_ = 4.88 × 10^-2^) (**Figure 3A**, **Supplementary Table 12**). All associations remained robust across sensitivity analyses for heterogeneity, pleiotropy, and causal directionality (**Supplementary Table 13**).

We further corroborated these findings in the UK Biobank cohort (*n* = 462,933). This analysis replicated the protective associations for PRKCQ (OR = 0.982; 95% CI: 0.975–0.988; FDR_IVW_ = 3.48 × 10^-7^) and C5 (OR = 0.997; 95% CI: 0.994–1.000; FDR_IVW_ = 2.4 × 10^-3^), identified an additional protective signal for ITGAM (OR = 0.976; 95% CI: 0.958–0.994; FDR_IVW_ = 1.92 × 10^-2^), and re-confirmed the risk-increasing effect of CD83 (OR = 1.006; 95% CI: 1.001–1.011; FDR_IVW_ = 2.43 × 10^-2^) (**Figure 3B**, **Supplementary Table 14**). Sensitivity analyses in this cohort likewise supported the reliability of these causal inferences (**Supplementary Table 15**).

To further disentangle causality from confounding by LD and pleiotropy, we performed SMR coupled with the HEIDI test. Critically, CD83, NFKB1, and PRKCQ all passed this stringent test (*P_SMR_* < 0.05 and *P_HEIDI_* > 0.05), providing strong evidence that the observed associations are driven by the expression of the genes themselves rather than by linked variants (**Figure 3C**, **Supplementary Table 16**).

Collectively, the consistent causal evidence across two independent cohorts and the rigorous SMR/HEIDI validation prioritized CD83 and PRKCQ for further therapeutic evaluation. The validated effects of C5, NFKB1, and ITGAM further highlighted a core set of genes for mechanistic and therapeutic evaluation in asthma.

### Convergent lines of evidence support CD83 as a candidate risk-associated pathway across asthma subtypes

**Figure 4:**
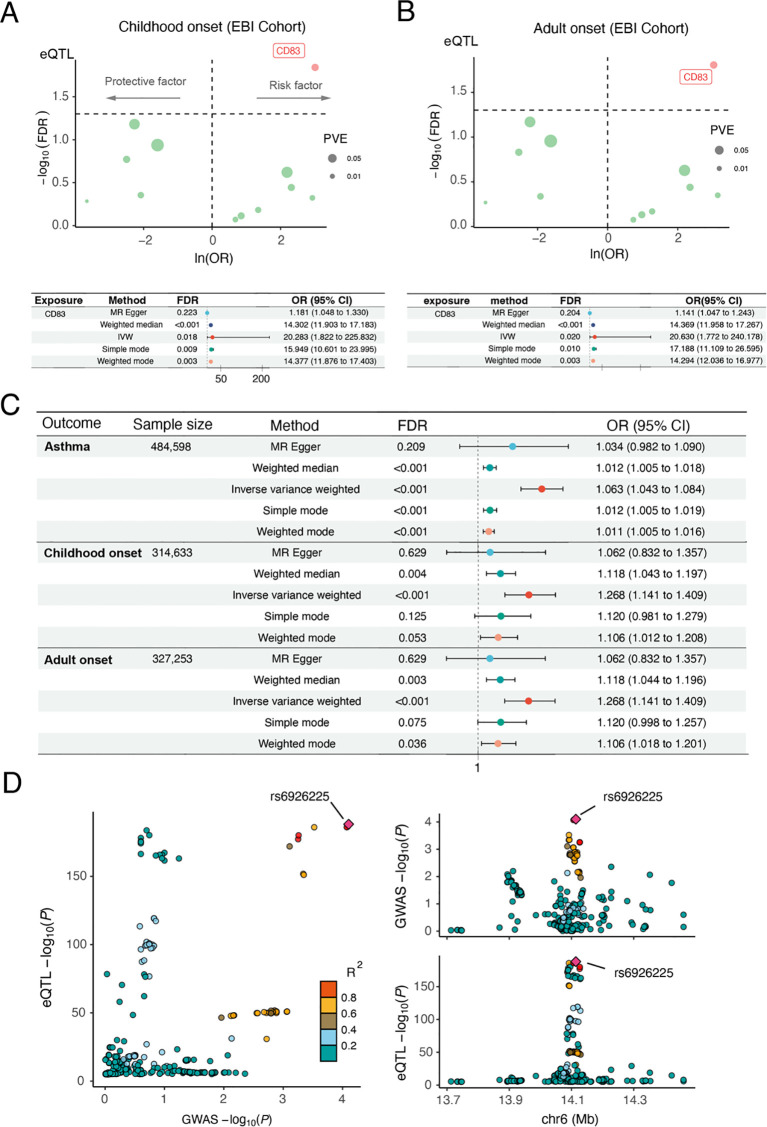
CD83 is consistently associated with asthma risk across subtypes and is supported by pQTL and colocalization analyses. **(A, B)** Mendelian randomization (MR) analysis of CD83 expression (*eQTL*) shows a consistent risk-increasing effect on **(A)** childhood-onset and **(B)** adult-onset asthma in the EBI cohort. Top panels are volcano plots showing the significance [-log_10_(FDR)] versus the effect size [ln(OR)]. Bottom panels are forest plots of the causal estimates from multiple MR methods. **(C)** MR analysis using genetically predicted CD83 protein levels (*pQTL*) supports a consistent risk-increasing association for overall asthma, as well as for childhood- and adult-onset subtypes. The forest plot shows odds ratios (OR) and 95% confidence intervals from five different MR methods. **(D)** Colocalization analysis provides strong evidence for a shared causal variant between the asthma GWAS signal and the *CD83* eQTL signal. (Left) LocusCompare plot showing a high correlation between GWAS *P*-values (-log_10_*P*) and *CD83* eQTL *P*-values (-log_10_*P*) for SNPs in the locus. Points are colored by linkage disequilibrium (R²) with the lead SNP (rs6926225). (Right) Regional association plots for asthma GWAS (top) and *CD83* eQTLs (bottom). The analysis yielded a high posterior probability of colocalization (PP.H4 = 0.89).

Given the heterogeneity of asthma, we performed subtype-specific MR analyses. Genetically predicted CD83 expression showed directionally consistent risk-increasing associations in childhood-onset (OR = 20.28; CI: 1.822, 225.832; FDR_IVW_ = 1.80 × 10^-2^), adult-onset (OR = 20.63; CI: 1.772, 240.178; FDR_IVW_ = 2.00 × 10^-2^), and pediatric asthma (OR = 1.068; CI: 1.010, 1.130; FDR_IVW_ = 2.44 × 10^-2^) (**Figures 4A, B**, **Supplementary Figure 2A**, **Supplementary Table 17**). Although all CD83 eQTL instruments were strong (F-statistics 36.22-859.02; **Supplementary Table 7**), the childhood-onset and adult-onset estimates had wide confidence intervals and should be interpreted as exploratory subtype evidence. In contrast, other candidate genes exhibited subtype-specific associations; for instance, C5 was primarily linked to allergic asthma, while CHI3L1 was predominantly associated with non-allergic asthma (**Supplementary Figures 2B, C**).

To determine if this causal effect extends from transcript to the functional protein, we conducted a pQTL-based MR analysis. The association was consistent and statistically robust: genetically predicted higher CD83 protein levels were linked to increased asthma risk (OR = 1.063; 95% CI: 1.043–1.084; FDR_IVW_ = 8.5×10^-10^) (**Figure 4C**, **Supplementary Tables 18-20**). This finding was consistent in both childhood- and adult-onset subtypes (**Figure 4C**), thereby outlining a coherent chain of evidence from gene transcription to protein abundance and ultimately to disease risk, strengthening the biological rationale for targeting CD83.

Finally, to confirm that the association signals for CD83 expression and asthma risk are driven by a shared causal variant, we performed colocalization analysis. Using the coloc (15, 16) R package, we found a high posterior probability of colocalization (PP.H4 = 0.892), providing strong support for a shared causal signal at the CD83 locus that is consistent with CD83 as the prioritized gene (**Figure 4D**, **Supplementary Table 21**). Consistent with these genetic findings, CD83 mRNA was also significantly upregulated in the nasal mucosa of asthma patients compared to healthy controls (**Supplementary Figure 2D**, *P* = 1.1 × 10^-5^). Together, these lines of evidence support CD83 as a candidate cross-subtype contributor to asthma.

### Cell-type-specific MR and cross-species scRNA-seq implicate APCs as the functional hub for CD83

**Figure 5:**
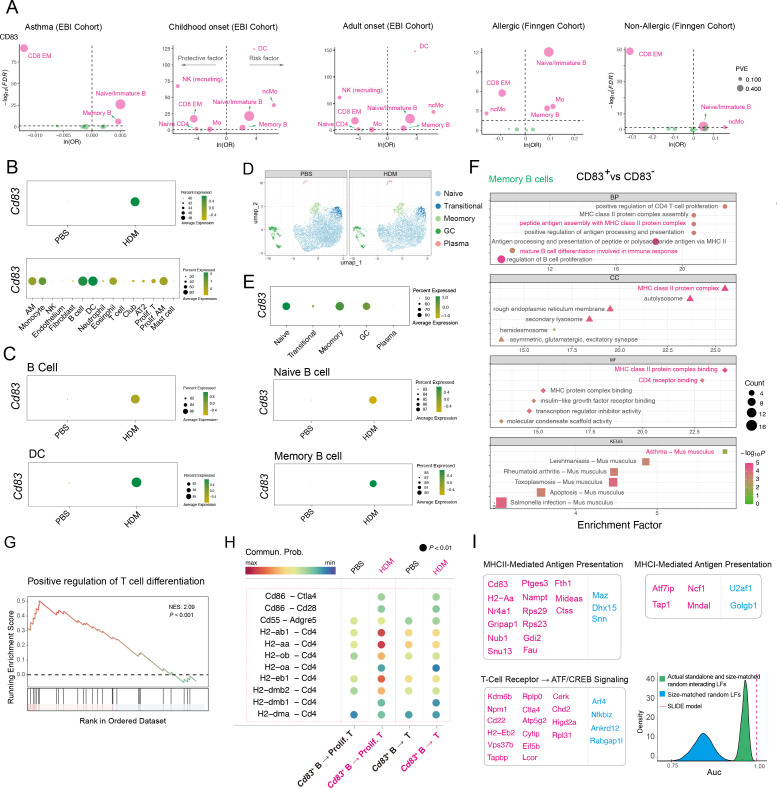
Cell-type-specific MR and mouse scRNA-seq identify CD83 in antigen-presenting cells as a potential driver of asthma. **(A)** Cell-type-specific Mendelian randomization (MR) volcano plots. The analysis links genetically predicted higher CD83 expression in specific immune cell types to asthma risk and its subtypes. CD83 expression in B cell subsets, dendritic cells (DCs), and non-classical monocytes (ncMo) is associated with increased risk, while expression in CD8^+^ effector memory (EM) T cells is protective. **(B)** Dot plots showing *Cd83* expression from scRNA-seq of whole lung tissue from a house dust mite (HDM)-induced mouse model of asthma. *Cd83* is upregulated after HDM challenge (top) and is primarily expressed in antigen-presenting cells (APCs) like B cells, DCs, and monocytes (bottom). AM: alveolar macrophage; AT2: alveolar type 2 cells; Prolif. T: Proliferating T cells. **(C)** Dot plots showing specific upregulation of *Cd83* in B cells and DCs after HDM challenge. **(D)** UMAP plots of B cell sub-clusters from the mouse model, showing populations of naïve, transitional, memory, germinal center (GC), and plasma cells in PBS versus HDM-challenged mice. **(E)** Dot plots showing that *Cd83* expression is highest in naïve and memory B cell subsets and is induced by HDM challenge. **(F)** Functional enrichment analysis of differentially expressed genes between *Cd83*^+^ and *Cd83*⁻ memory B cells. Results show significant enrichment in pathways related to MHCII-mediated antigen presentation and T cell proliferation. **(G)** Gene Set Enrichment Analysis (GSEA) plot showing that the “Positive regulation of T cell differentiation” gene set is significantly enriched in *Cd83*^+^ memory B cells. **(H)** Cell-cell communication analysis (CellChat) showing enhanced interaction probability between *Cd83*^+^ B cells and T cells via the MHC-II pathway (e.g., H2-aa – Cd4) in HDM-challenged mice. **(I)** Identification of the core mechanism in Cd83^+^ memory B cells using the SLIDE machine learning algorithm. (Left) Key genes identified as context-specific biomarkers for the MHC-II-mediated antigen presentation pathway. (Right) Density plot showing the performance (Area Under the Curve, AUC) of the SLIDE model built with the actual significant latent factors (green distribution and dashed red line) compared to null distributions from models built with size-matched random factors (blue). The superior performance of the actual model validates the significance of the identified mechanism.

To deconstruct the cellular origins of CD83’s pro-asthmatic effects, we performed cell-type-specific MR by integrating single-cell eQTL data. Genetically predicted higher CD83 expression in B-cell subsets and DCs was consistently associated with increased asthma risk, whereas the estimates for non-classical monocytes varied across asthma subtypes. CD83 expression in effector-memory CD8^+^ T cells showed a protective association (**Figure 5A**, **Supplementary Table 22**). These findings from human causal genetics support B cells and other APCs as a likely cellular context for CD83-associated asthma risk.

To corroborate this genetic hypothesis in a pathophysiological context, we performed scRNA-seq on lung tissue from an HDM-challenged mouse model (**Supplementary Figure 3**). Consistent with our human genetic findings, HDM challenge not only upregulated overall CD83 expression but also enriched its expression specifically within B cells, DCs, and monocytes (**Figures 5B, C**,). This *in vivo* model thus provided a powerful platform to dissect the CD83-associated transcriptional programs in these key APCs.

A detailed sub-analysis of these APCs revealed a convergent mechanism. In B cells, CD83 expression was highest in the naïve and memory subsets and was induced by HDM (**Figures 5D, E**). A suite of computational analyses—including functional enrichment, Gene Set Enrichment Analysis (GSEA), and CellChat—demonstrated that CD83^+^ memory B cells were enhanced for MHCII antigen presentation and interaction with T cells (**Figures 5F–H**). This central mechanism was further validated by the SLIDE (41), a machine-learning framework for identifying latent biological factors and context-specific biomarkers, which identified the MHCII pathway as the most significant latent factor distinguishing the CD83^+^ cell state (**Figure 5I**). Critically, parallel analyses in DCs revealed a conserved association, with CD83 expression similarly linked to the enrichment of antigen-presentation programs (**Supplementary Figure 3D, E**).

We further examined whether the CD83-positive DC state was linked to APC activation in the HDM model. After HDM challenge, lung DCs showed increased expression of Cd83 together with activation- and antigen-presentation-related markers, including Cd86, Cd80, Cd40, H2-Ab1, and Cd74 (**Supplementary Figure 3F, G**). At the single-cell level, Cd83 expression in DCs was positively correlated with Cd86, Cd40, and Cd80 expression under both PBS and HDM conditions (**Supplementary Figure 3H**). As a focused functional validation, CD11c promoter-driven shRNA-mediated Cd83 knockdown reduced Cd83 expression in lung DCs and decreased DC CD86 expression after HDM exposure (**Supplementary Figure 3I**). These data support the biological relevance of the CD83-positive DC activation state identified by the genetic and single-cell analyses, while leaving the detailed molecular mechanism for future investigation.

**Figure 6:**
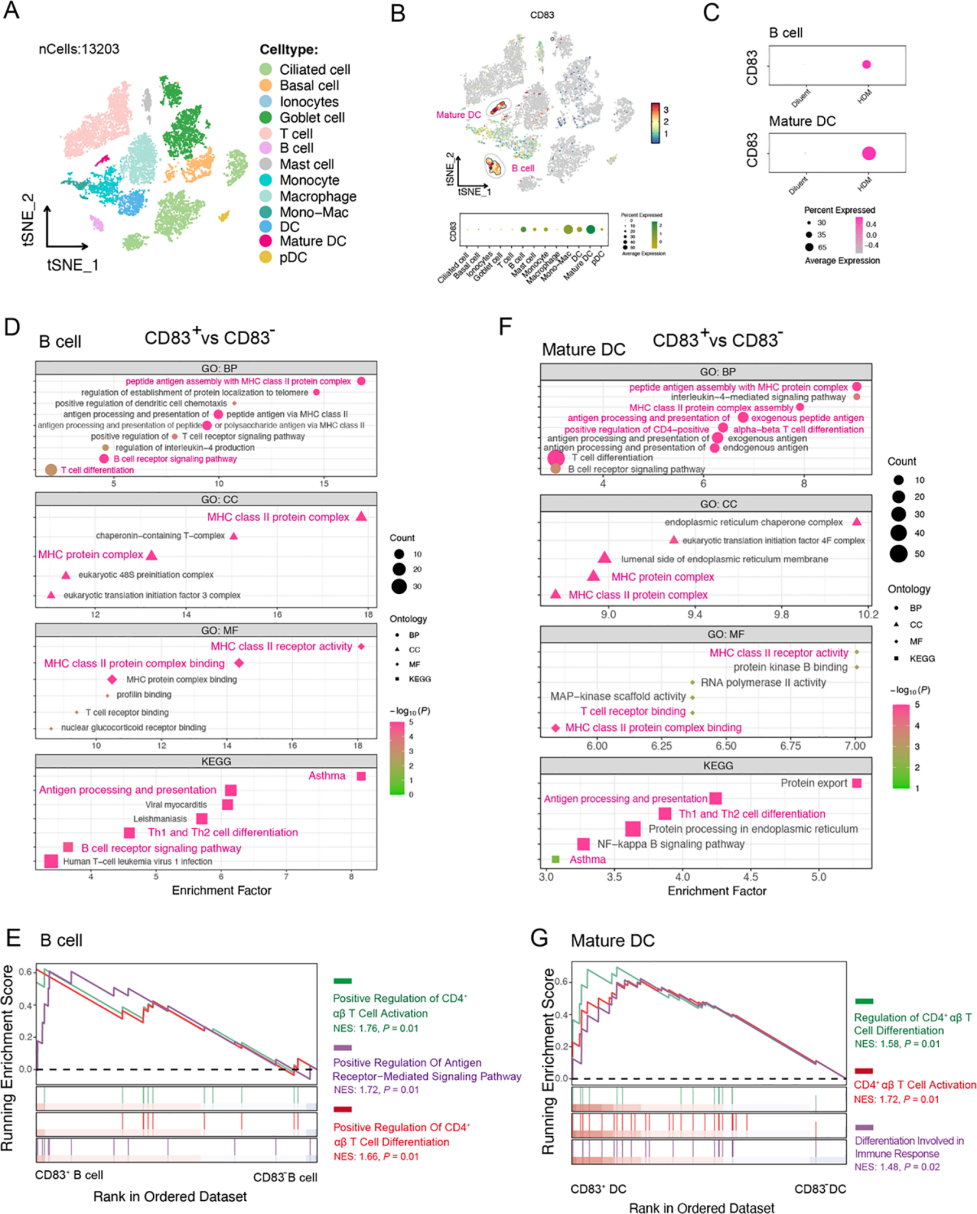
Single-cell analysis of human bronchial brushings reveals allergen-induced CD83 expression and a conserved antigen presentation mechanism. **(A)** t-SNE plot showing the major cell populations identified from single-cell analysis of bronchial brushings from asthma patients (*n* = 3 subjects; *n*Cells = 13,203; data from GSE164015). Samples were collected before (diluent) and after (house dust mite; HDM) allergen challenge. **(B)** Feature plot (top) and dot plot (bottom) showing that CD83 expression is predominantly localized to B cells and mature dendritic cells (DCs). **(C)** Dot plots showing significant upregulation of CD83 expression in B cells and mature DCs after *in vivo* HDM allergen challenge compared to the pre-challenge (diluent) baseline within the same subjects. **(D, F)** Functional enrichment analysis of differentially expressed genes between *CD83*^+^ and *CD83*⁻ cells for **(D)** B cells and **F,** mature DCs. In both cell types, CD83 expression is associated with significant enrichment of pathways related to MHCII antigen presentation and T cell differentiation. **(E, G)** Gene Set Enrichment Analysis (GSEA) plots showing that gene sets for CD4^+^ T cell activation and differentiation are significantly enriched in *CD83*^+^ cells compared to *CD83*⁻ cells in both **(E)** B cells and **(G)** mature DCs.

Finally, cross-species analyses support conservation of this mechanistic axis, with CD83-positive APCs enriched for MHC class II antigen-presentation programs. In bronchial brushings from asthma patients undergoing allergen challenge (data from GSE164015 (42)), we confirmed that CD83 was upregulated in B cells and DCs (**Figures 6A–C**, **Supplementary Figure 4**) and associated with an antigen-presentation signature (**Figures 6D–G**). This finding was replicated in lung tissue from a cynomolgus monkey asthma model (data from GSE213085 (43)), which also showed CD83 expression in B cells and DCs linked to T cell activation pathways (**Supplementary Figure 5**). This cross-species consistency supports the translational relevance of the CD83-associated APC program in asthma.

### *In silico* drug screening and phenome-wide analysis nominate candidate strategies for modulating the CD83 pathway

**Figure 7:**
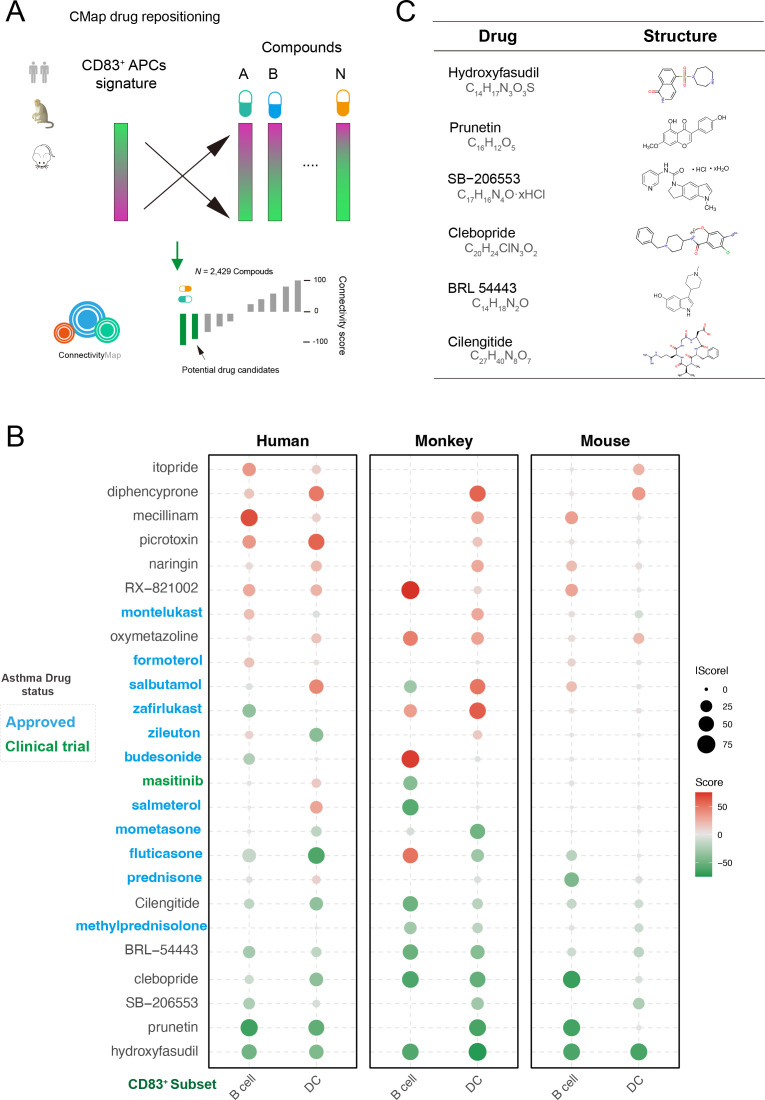
*In-silico* drug screen highlights candidate compounds predicted to reverse the CD83^+^ APC signature. **(A)** Schematic of the Connectivity Map (CMap) drug repositioning strategy. The transcriptional signatures derived from *CD83*^+^ B cells and DCs across human, monkey, and mouse were used to query the CMap database of 2,429 compounds. Compounds are ranked by a connectivity score, with highly negative scores indicating a strong ability to reverse the disease signature. **(B)** Dot plot showing the connectivity scores for selected compounds against the *CD83*^+^ B cell and DC signatures from human, monkey, and mouse. Green indicates reversal of the pathogenic signature (negative score), while red indicates potentiation (positive score). The analysis identifies several candidate compounds (e.g., hydroxyfasudil) and provides mechanistic insights into existing asthma therapies. **(C)** Chemical structures of the top-ranked candidate compounds identified in the screen.

To identify potential therapeutics modulating the CD83-associated APC state, we performed an *in-silico* screen using the CMap (20, 46) database. We queried 2,429 compounds for their ability to reverse the pathogenic transcriptional signatures derived from cross-species CD83^+^ B cells and DCs (**Figure 7A**). This screen prioritized hydroxyfasudil (the active metabolite of fasudil) and prunetin as top candidates capable of robustly reversing the pathogenic signature (**Figures 7B, C**). Both compounds have known anti-inflammatory properties, positioning them as candidate compounds for future experimental evaluation (47, 48).

Notably, our unbiased approach successfully identified several approved corticosteroids—including methylprednisolone, prednisone, and fluticasone—as potent reversers of the CD83^+^ cell signature (**Figure 7B**). This finding supports the biological relevance of the signature and suggests that suppression of the CD83-APC program may contribute to corticosteroid action. In contrast, several other widely used asthma drugs, including montelukast and formoterol, showed positive connectivity scores (**Figure 7B**), indicating similarity rather than reversal of the queried transcriptional program. Masitinib, a kinase inhibitor evaluated clinically for severe asthma, also showed strong reversal of the monkey CD83^+^ B-cell signature (**Figure 7B**). These observations generate testable hypotheses regarding differential pathway modulation in asthma.

Having identified candidate compounds, we next assessed the broader specificity of the CD83 signal. We performed a PheWAS analysis using CD83 eQTLs as genetic proxies to systematically evaluate the potential effects of lifelong CD83 modulation on thousands of human phenotypes using the PheWAS Portal and PheWeb databases (19, 45). The analysis revealed no significant association between genetically predicted CD83 expression and any other clinical phenotype at a genome-wide significance threshold (*P* < 5 × 10^-8^) (**Supplementary Figure 6**, **Supplementary Table 23**). This result is reassuring with respect to target specificity but does not by itself establish the safety of pharmacologic CD83 inhibition.

## Discussion

In this study, we used a multi-omic causal inference framework to prioritize CD83 as a genetically supported candidate therapeutic target in asthma. Across independent datasets, higher genetically predicted CD83 expression was consistently associated with increased asthma risk, and this signal was supported at the protein level. By integrating colocalization, SMR/HEIDI, cell-type-specific genetic analyses, and cross-species single-cell transcriptomics, we further localized this association to an antigen-presenting-cell-centered program, particularly in B cells and dendritic cells.

A major strength of the study is that it moves beyond locus discovery alone. Many asthma-associated genes emerge from genetic studies, but relatively few can be connected to a plausible cellular context and therapeutic hypothesis. In our analysis, CD83 remained consistently prioritized across discovery and replication datasets and retained support across complementary approaches designed to reduce confounding by linkage disequilibrium and pleiotropy. This convergence provides stronger support for CD83 than is typically available for candidate genes identified from association signals alone.

Our results also place CD83 in a biologically coherent context. CD83 is already recognized as an activation-associated molecule in antigen-presenting cells and has been linked to regulation of MHC class II and co-stimulatory pathways (46–52). In that sense, the present findings are biologically plausible. The additional contribution of this study is to connect that established immunology to asthma and to suggest that the relevant signal is concentrated in specific APC populations rather than distributed broadly across the immune system. In particular, the consistent enrichment of antigen-presentation and T-cell-activation programs in CD83-positive B cells and DCs across human, mouse, and monkey datasets supports an APC-centered interpretation of the genetic signal.

This cell-specific context is important because it suggests a more focused mechanistic hypothesis. Rather than interpreting CD83 simply as a general marker of immune activation, our data support a model in which CD83 is associated with an APC state that is permissive for enhanced antigen presentation and downstream T-cell interaction during allergic airway inflammation. The consistency of this pattern across species and analytical platforms strengthens the robustness of this interpretation. At the same time, these findings remain inferential. Although the combined genetic and transcriptomic evidence is strong, direct perturbation studies will still be required to determine whether CD83 itself is necessary for this APC program and whether modulating CD83 can alter disease-relevant airway inflammation.

Another notable aspect of the study is the apparent context dependence of CD83. In our cell-type-specific analyses, higher CD83 expression in APC populations was associated with increased asthma risk, whereas expression in effector-memory CD8-positive T cells appeared protective. This observation is consistent with the broader literature (49–56) suggesting that CD83 can have distinct functions across immune compartments, including regulatory roles in the lung (57). Taken together, these results suggest that the net association of CD83 with asthma risk may reflect the predominance of its APC-associated effects at the population level, underscoring the need for therapeutic strategies that preferentially modulate APC-associated CD83 programs rather than broadly inhibiting CD83 systemically.

The translational implications are encouraging but should be interpreted cautiously. The phenome-wide analysis did not identify major genome-wide significant associations outside the asthma signal, which is reassuring with respect to broad pleiotropic liability, but this does not establish the safety of pharmacologic inhibition. Similarly, CMap identified compounds predicted to reverse the CD83-positive APC transcriptional program, including hydroxyfasudil and prunetin; however, these findings are hypothesis-generating and should not be interpreted as evidence of direct CD83 inhibition or established therapeutic efficacy. The identification of corticosteroids as negative-connectivity compounds is nonetheless informative, because it supports the biological relevance of the queried signature and suggests that suppression of this APC-associated program may contribute to established therapy.

Several limitations should be acknowledged. First, the main human genetic analyses were conducted primarily in populations of European ancestry, which may limit generalizability. Second, the eQTL and pQTL instruments were derived largely from blood-based datasets and may not fully capture airway compartment biology. Third, some subtype-specific estimates were large and imprecise and should therefore be interpreted cautiously. Fourth, FOLR2 showed a strong discovery-stage association but was not prioritized for mechanistic follow-up because CD83 had broader convergent support across validation layers (**Supplementary Table 24**). Finally, the focused DC-specific CD83 knockdown experiment supports the biological relevance of CD83 in APC activation and HDM-induced airway inflammation, but it does not provide a complete mechanistic explanation. Future studies are needed to define the necessity, sufficiency, and downstream molecular mechanisms of CD83 in APC antigen presentation and T-cell activation.

## Conclusions

Overall, our findings prioritize CD83 as a genetically supported candidate therapeutic target in asthma and suggest that its disease relevance is most likely mediated through antigen-presenting cells, particularly B cells and DCs. By linking human causal genetics with cell-specific and cross-species transcriptomic evidence, this study provides a rationale for direct CD83 perturbation studies in airway inflammation models.

## Data Availability

The datasets presented in this study can be found in online repositories. The names of the repository/repositories and accession number(s) can be found below: CNP0007739 (https://db.cngb.org/data_resources/?query=CNP0007739).
