# Supplementary material for: Genetic and cross-species single-cell analyses prioritize CD83 in antigen-presenting cells as a candidate therapeutic target in asthma
Source: Front Immunol. 2026 Jul 13;17:1859076. doi: 10.3389/fimmu.2026.1859076 (PMC13402173; doi:10.3389/fimmu.2026.1859076)
Supplement: Supplementary file 2 [file DataSheet2.zip › Supplemental Materials/Supplementary Figures_Revised.docx]

**Supplementary Figures and Figure legends:**


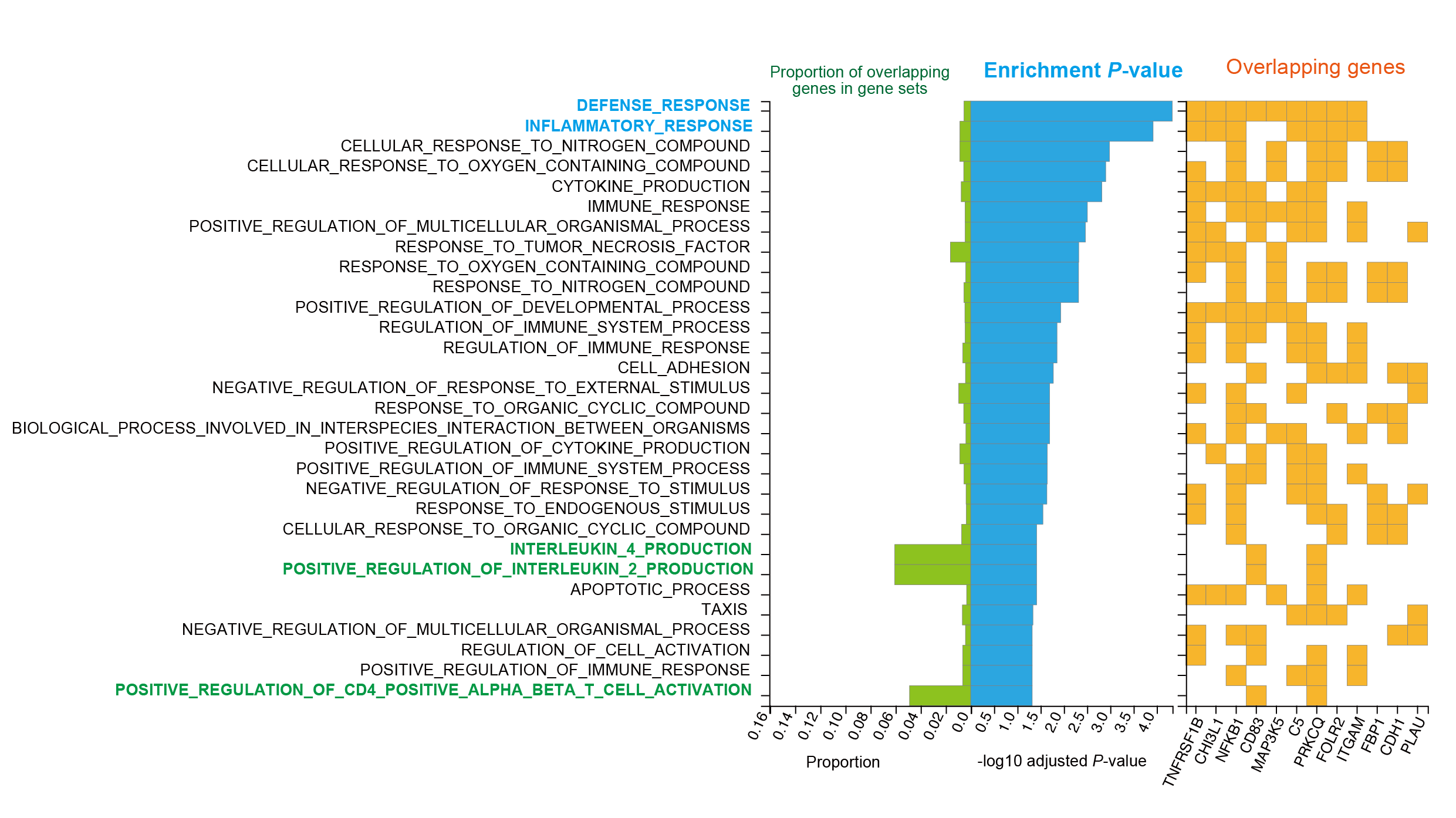


**Fig. S1. Functional enrichment analysis of the 12 candidate genes.**
Gene Ontology (GO) enrichment analysis for Biological Process terms was performed on the 12 prioritized candidate genes. The plot displays the top enriched pathways, ranked by significance. For each pathway (y-axis), the plot shows: (**left, green**) the proportion of genes from the pathway present in our 12-gene set; (**middle, blue**) the enrichment significance as -log_10_(adjusted *P*-value); and (**right, gold matrix**) the specific candidate genes (x-axis) that are members of that pathway. The analysis reveals significant enrichment in pathways central to asthma pathophysiology, including inflammatory/defense responses, cytokines (IL-4/IL-2) production, and T-cell activation (highlighted in blue and green text).


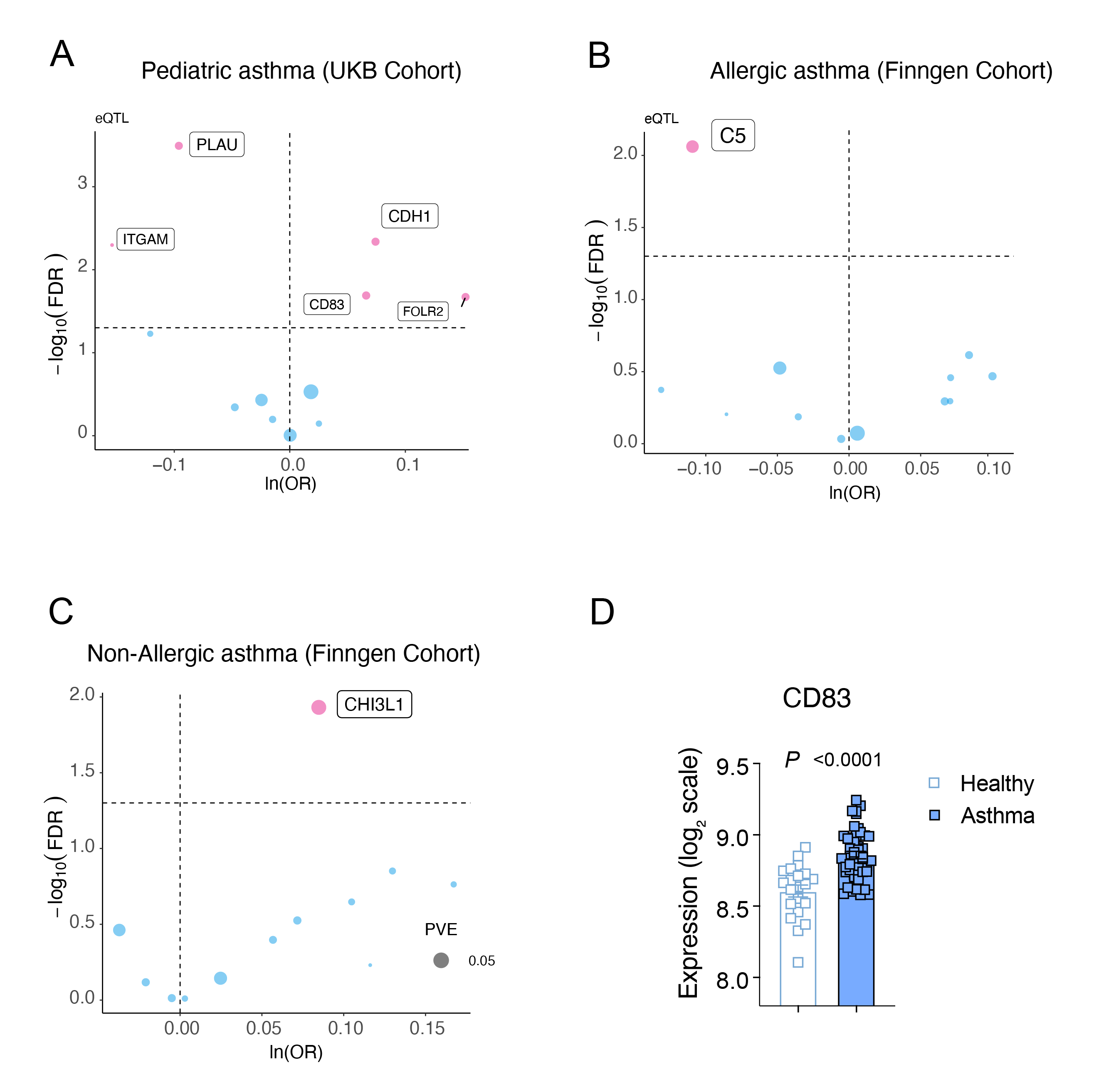


**Fig. S2. Subtype-specific MR analyses and differential expression of** **CD83.**
**A-C:** Mendelian randomization (MR) volcano plots showing causal associations between genetically predicted gene expression (eQTL) and risk for specific asthma subtypes. Plots show results for **A,** Pediatric asthma (UKB Cohort);  **B,** Allergic asthma (FinnGen Cohort); and **C,** Non-Allergic asthma (FinnGen Cohort). The x-axis represents the effect size [ln(OR)] and the y-axis shows the significance [-log_10_(FDR)]. Genes with significant associations (FDR < 0.05, dashed line) are labeled. Pink points indicate genes passing the significance threshold.
**E:** Box and jitter plot showing significantly higher *CD83* mRNA expression in nasal mucosal tissue from individuals with asthma compared to healthy controls (*P* < 0.0001, two-sided *t*-test). Each point represents an individual sample.


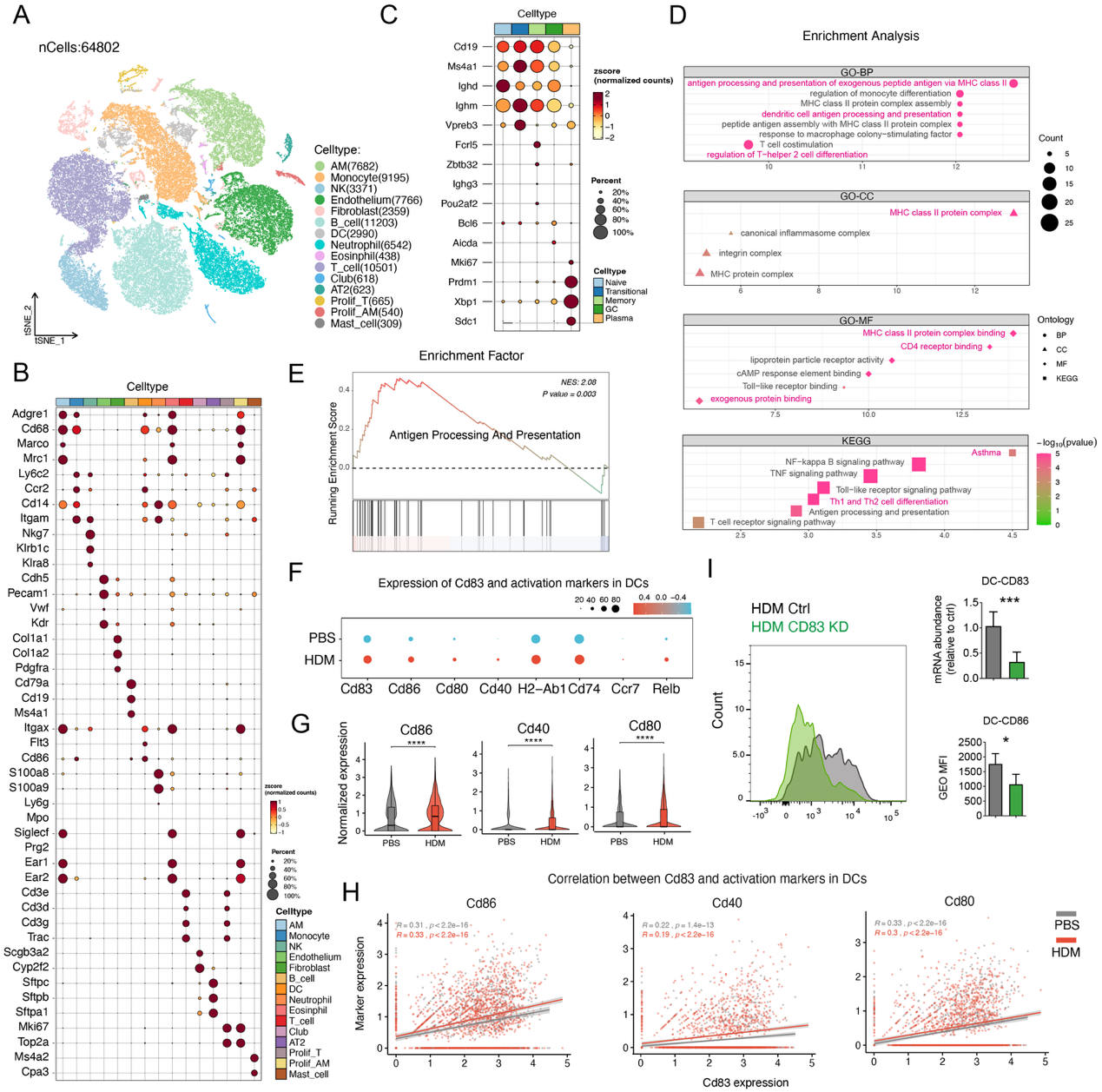


**Fig. S3. Single-cell and functional validation of CD83-associated dendritic-cell activation in an HDM-challenged mouse model.**
**A:** t-SNE plot showing the major cell populations identified in the lungs of control and HDM-challenged mice (nCells = 64,802). Cell types are annotated and colored as indicated. AM: alveolar macrophage; AT2: alveolar type 2 cells; Prolif. T: Proliferating T cells.
**B:** Dot plot showing the expression of canonical marker genes used to identify the major cell types shown in (A). Dot size represents the percentage of cells expressing the gene; color indicates the average expression level.
**C:** Dot plot showing the expression of canonical marker genes used to annotate the B cell sub-clusters (Naïve, Transitional, Memory, GC, Plasma).
**D:** Functional enrichment analysis of differentially expressed genes in dendritic cells (DCs) comparing CD83⁺ versus CD83⁻. Results show significant enrichment in pathways related to antigen processing and presentation via MHC class II.
**E:** Gene Set Enrichment Analysis (GSEA) plot showing that the "Antigen Processing And Presentation" gene set is significantly enriched in CD83⁺ DCs (NES = 2.08, *P* = 0.003).

**F:** Dot plot showing the expression of Cd83 and activation- or antigen-presentation-related markers in lung DCs from PBS- and HDM-treated mice.

**G:** Violin plots showing increased expression of Cd86, Cd40, and Cd80 in lung DCs after HDM challenge.

**H:** Correlation analysis showing positive associations between Cd83 expression and Cd86, Cd40, or Cd80 expression in lung DCs under PBS and HDM conditions.

**I:** CD11c promoter-driven shRNA-mediated Cd83 knockdown reduced Cd83 mRNA abundance in lung DCs and decreased CD86 expression on DCs after HDM exposure.


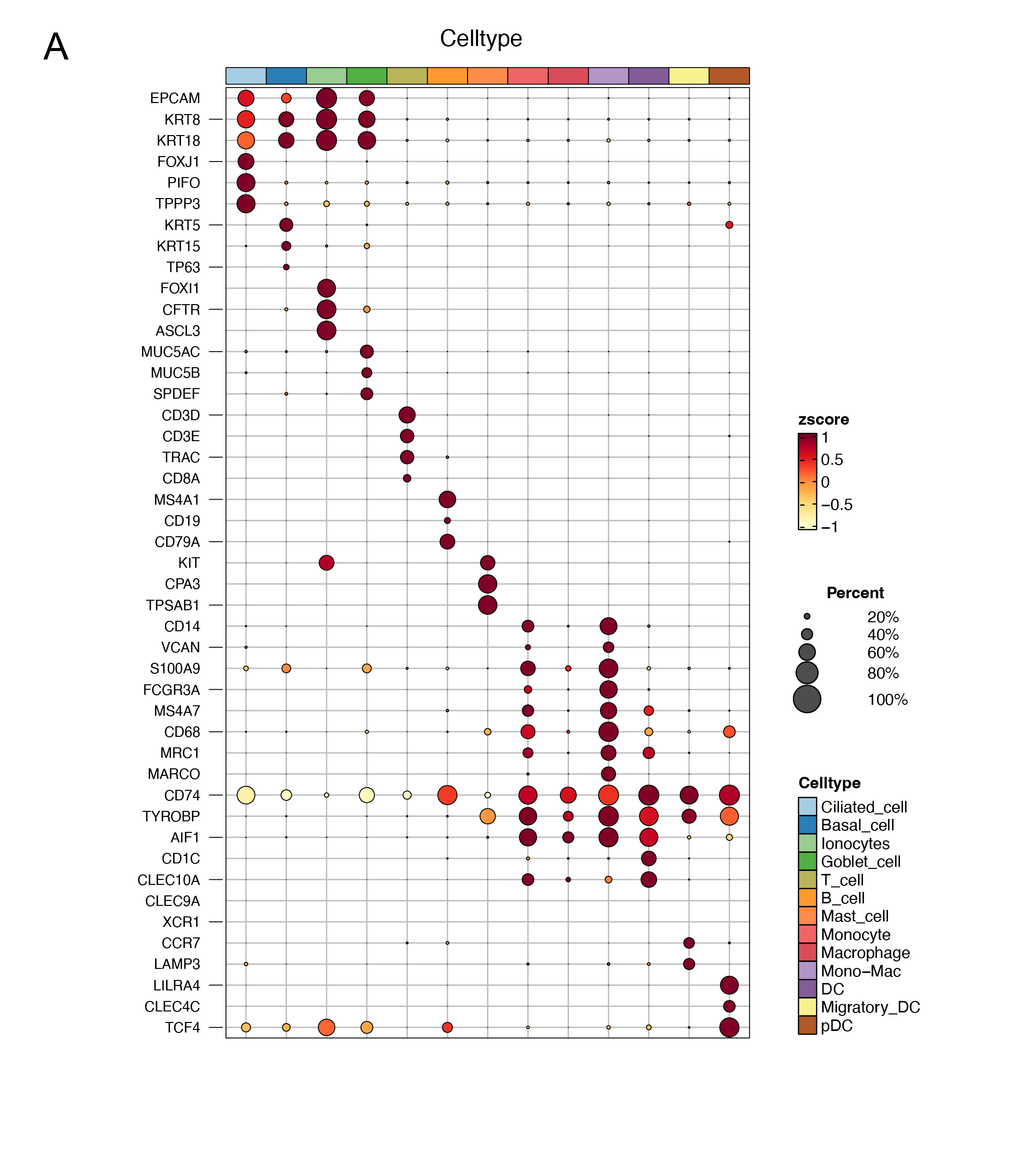


**Fig. S4. Expression of canonical marker genes used for cell type annotation in human bronchial brushings.**
**A:** Dot plot showing the expression of canonical marker genes used to identify the major cell populations from the scRNA-seq analysis of human bronchial brushings (data from GSE164015), corresponding to the clusters shown in Fig. 6A. Dot size represents the percentage of cells within a cluster expressing the gene, and color indicates the average z-scored expression level.

**
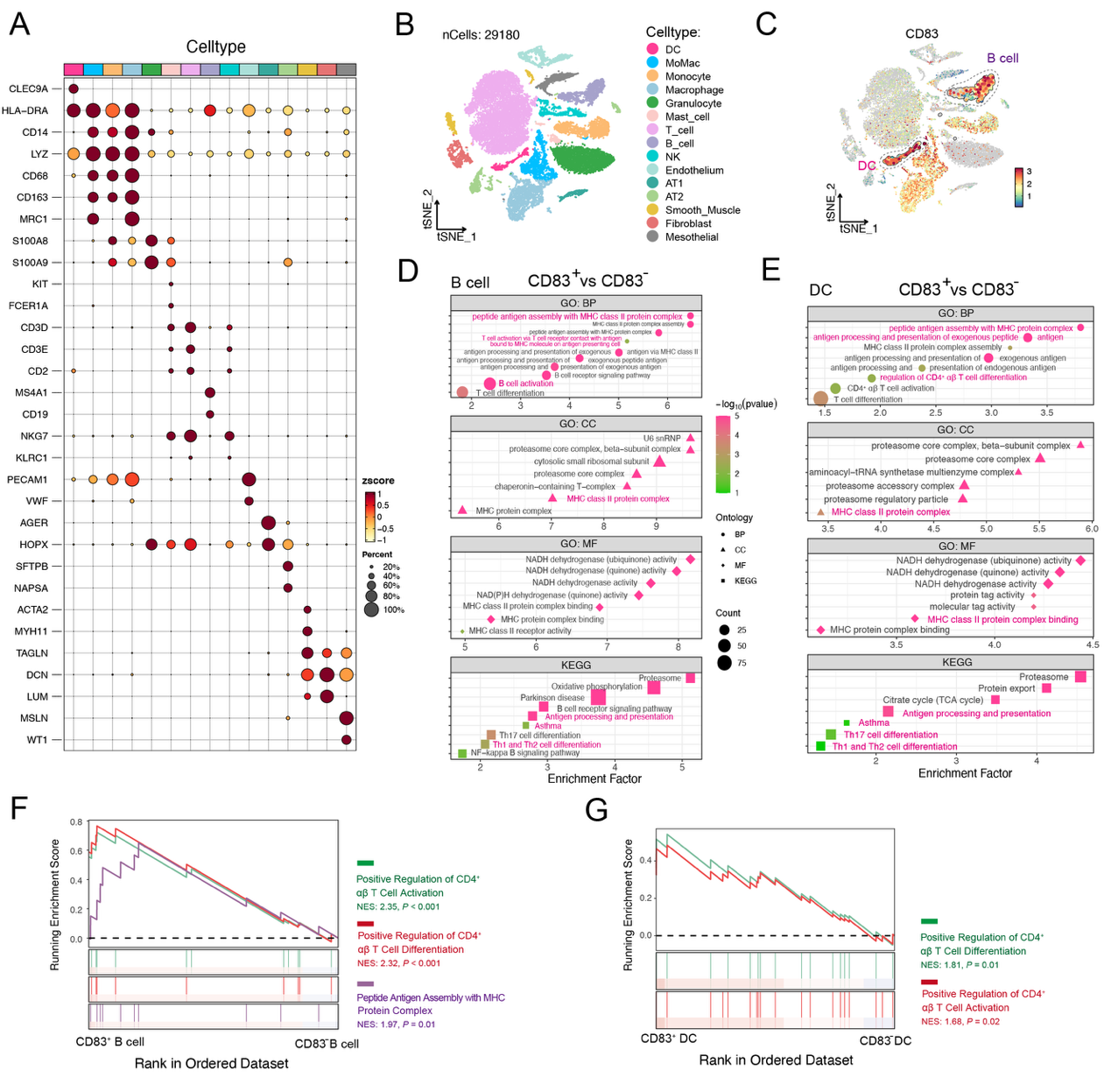
Fig. S5. Conservation of the CD83-driven antigen presentation mechanism in a cynomolgus monkey model of asthma.**
scRNA-seq analysis of lung tissue from an *Ascaris suum*-induced asthma model in cynomolgus monkeys (data from GSE213085).
**A:** Dot plot showing the expression of canonical marker genes used for cell type annotation.
**B:** t-SNE plot showing the major cell populations identified (nCells = 29,180).
**C:** Feature plot showing that *CD83* expression is predominantly localized to B cells and DCs.
**D,E:** Functional enrichment analysis of differentially expressed genes between *CD83*⁺ and *CD83*⁻ cells for **D,** B cells and **E,** DCs. In both cell types, *CD83* expression is associated with significant enrichment of pathways related to MHC class II antigen presentation and T cell differentiation.
**F,G:** Gene Set Enrichment Analysis (GSEA) plots showing that gene sets for T cell activation and differentiation are significantly enriched in *CD83*⁺ cells compared to *CD83*⁻ cells in both **F,** B cells and **G,** DCs.


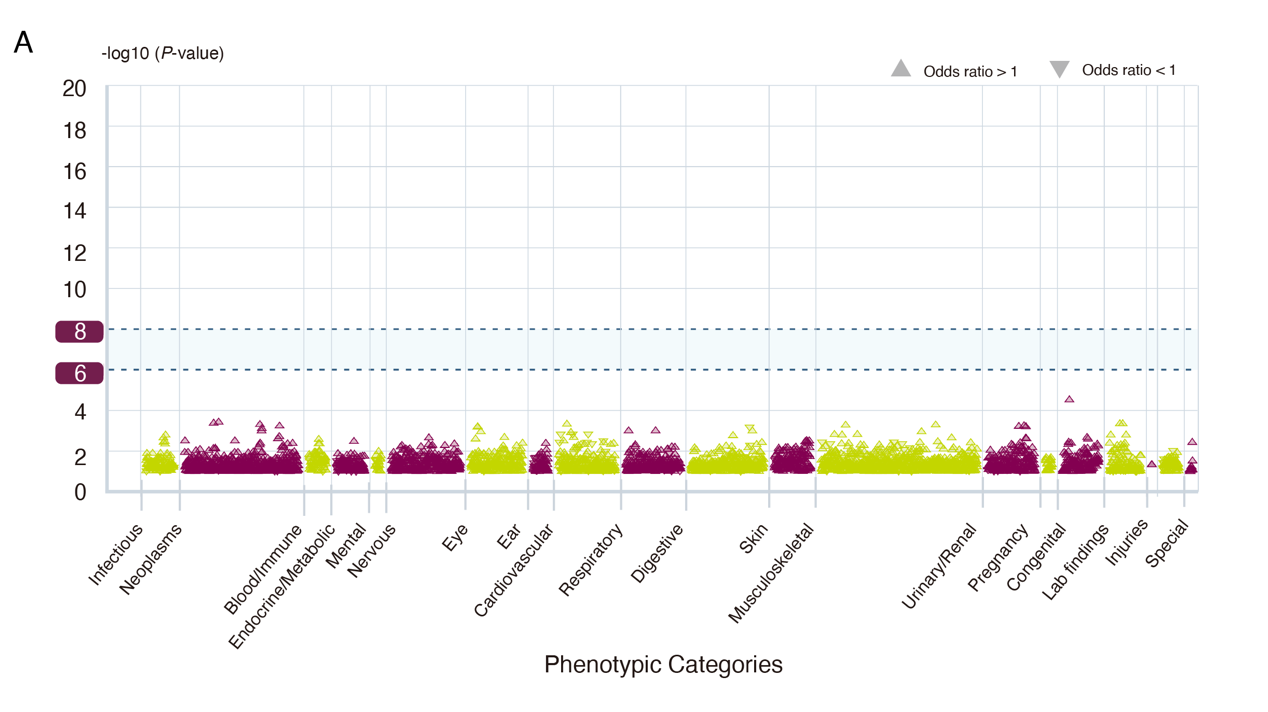


**Fig. S6. Phenome-wide safety analysis of CD83 reveals a low risk of systemic adverse effects.**
**A:** Phenome-wide association study (PheWAS) plot showing the causal association of genetically predicted *CD83* expression with thousands of clinical phenotypes, grouped by category. The y-axis represents the association significance [-log10(*P*-value)]. Each point is a phenotype, with upward triangles indicating risk-increasing effects (OR > 1) and downward triangles indicating protective effects (OR < 1). The top dashed line indicates the conventional genome-wide significance threshold (*P*< 5 × 10⁻⁸). No phenotype reached this significance level, supporting a favorable on-target safety profile for therapeutic CD83 modulation.
