# Supplementary material for: Genetic and cross-species single-cell analyses prioritize CD83 in antigen-presenting cells as a candidate therapeutic target in asthma
Source: Front Immunol. 2026 Jul 13;17:1859076. doi: 10.3389/fimmu.2026.1859076 (PMC13402173; doi:10.3389/fimmu.2026.1859076)
Supplement: Supplementary file 2 [file DataSheet2.zip › Supplemental Materials/Supplemental Materials.docx]

**Supplemental material**

Fig. S1. Functional enrichment analysis of the 12 candidate genes.

Fig. S2. Subtype-specific MR analyses and differential expression of CD83.

Fig. S3. Single-cell and functional validation of CD83-associated dendritic-cell activation in an HDM-challenged mouse model.

Fig. S4. Expression of canonical marker genes used for cell type annotation in human bronchial brushings.

Fig. S5. Conservation of the CD83-driven antigen presentation mechanism in a cynomolgus monkey model of asthma.

Fig. S6. Phenome-wide safety analysis of CD83 reveals a low risk of systemic adverse effects.

Table S1. STROBE-MR Checklist for Mendelian Randomization Studies.

Table S2. Data Sources Used in the Study.

Table S3. List of potential drug target genes identified using the Drug-Gene Interaction Database.

Table S4. Comprehensive listing of potential drug target genes derived from the study conducted by Finan et al.

Table S5. Identified overlapping druggable genes from the Drug-Gene Interaction Database and Finan et al.’s study.

Table S6. Catalog of common druggable genes employed in this study.

Table S7. Summary of genetic variants of expression Quantitative Trait Loci (eQTLs) utilized during the discovery phase of this study.

Table S8. Results of Mendelian Randomization (MR) analysis following *P*-value filtration during the discovery phase of the study.

Table S9. Results of heterogeneity tests conducted during the discovery phase of this study.

Table S10. Results of the MR Egger intercept test for horizontal pleiotropy conducted during the discovery phase of this study.

Table S11. Results of the Steiger test conducted during the discovery phase of this study to assess the directionality of causation in genetic associations.

Table S12. Detailed results of Mendelian randomization analyses conducted in the replication phase using the European Bioinformatics Institute (EBI) cohort.

Table S13. Results of the sensitivity analysis and steiger testing conducted in the replication phase using the European Bioinformatics Institute (EBI) cohort.

Table S14. Results of Mendelian randomization analyses in the replication phase using the UK Biobank (UKB) cohort.

Table S15. Detailed results from the sensitivity analysis and steiger testing conducted in the replication phase using the UK Biobank (UKB) cohort.

Table S16. Results of Mendelian randomization analyses performed in the replication phase for asthma subtypes.

Table S17. Results of Summary-data-based Mendelian Randomization (SMR) and Heterogeneity In Dependent Instruments (HEIDI) tests evaluating the association between CD83 gene expression levels and asthma.

Table S18. Catalog of genetic variants identified as protein Quantitative Trait Loci (pQTLs) of CD83.

Table S19. Results of Mendelian randomization analyses investigating the causal relationship between protein levels of CD83 and asthma susceptibility.

Table S20. Sensitivity analysis results for the protein Quantitative Trait Loci (pQTL) Mendelian Randomization (MR) analysis.

Table S21: Colocalization analysis results of expression Quantitative Trait Loci (eQTL) for the CD83 gene with Single Nucleotide Polymorphisms (SNPs) associated with asthma.

Table S22. Single‑cell eQTL Mendelian randomization results for asthma and its subtypes.

Table S23. Phenome-wide association study (PheWAS) results for CD83 based on data extracted from the PheWeb database.

Table S24. Comparative evidence supporting prioritization of CD83 over discovery-stage candidate genes.
